# MicroarrayDesigner: an online search tool and repository for near-optimal microarray experimental designs

**DOI:** 10.1186/1471-2105-10-304

**Published:** 2009-09-22

**Authors:** Ahmet Sacan, Nilgun Ferhatosmanoglu, Hakan Ferhatosmanoglu

**Affiliations:** 1 Computer Engineering Department, Middle East Technical University, Ankara, Turkey; 2 Department of Computer Science and Engineering, The Ohio State University, Columbus, OH, USA; 3 Department of Industrial and Systems Engineering, The Ohio State University, Columbus, OH, USA

## Abstract

**Background:**

Dual-channel microarray experiments are commonly employed for inference of differential gene expressions across varying organisms and experimental conditions. The design of dual-channel microarray experiments that can help minimize the errors in the resulting inferences has recently received increasing attention. However, a general and scalable search tool and a corresponding database of optimal designs were still missing.

**Description:**

An efficient and scalable search method for finding near-optimal dual-channel microarray designs, based on a greedy hill-climbing optimization strategy, has been developed. It is empirically shown that this method can successfully and efficiently find near-optimal designs. Additionally, an improved interwoven loop design construction algorithm has been developed to provide an easily computable general class of near-optimal designs. Finally, in order to make the best results readily available to biologists, a continuously evolving catalog of near-optimal designs is provided.

**Conclusion:**

A new search algorithm and database for near-optimal microarray designs have been developed. The search tool and the database are accessible via the World Wide Web at . Source code and binary distributions are available for academic use upon request.

## Background

Microarray experiments are commonly used to detect differential expression of genes across a number of conditions of interest. In a typical two-color microarray experiment, cDNA *varieties *(also denoted as *treatments *or *samples*) from two experimental conditions are labeled with two different fluorophores (e.g., Cy3 green and Cy5 red fluorescent dyes), and hybridized onto the same slide of complementary probes. Relative intensities of each fluorophore is then used to quantify differential expression levels of the genes from the two treatments.

The data generated by microarray experiments are highly multidimensional and contain a considerable amount of noise due to variability associated with slide preparation and measurement. Therefore, careful planning is required in order to obtain statistically significant and biologically valid conclusions [1]. Theoretical experimental design studies aim to identify, in advance, the expected accuracy of the results that can be obtained from the microarray experiments (see [2] for a recent survey). Several evaluation criteria have been proposed in order to quantify the optimality of a given experimental design, with A, L, and D-optimality being the most widely used criteria [3,4].

**Figure 1:**
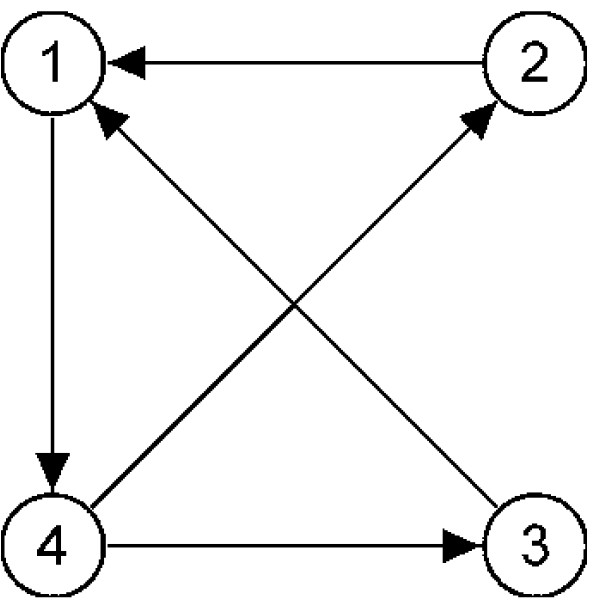
**Sample experimental design**. An experimental design with 4 varieties (vertices) and 5 microarray slides (edges) can be represented as a directed graph. The direction of the edges specify the color assignment of the varieties on a slide (e.g., from green to red).

An experimental design can be represented as a directed graph as shown in Figure 1. The vertices in the graph represent different experimental conditions or time points that cDNA varieties are obtained from, and each edge represents a single microarray slide. The direction of the edges specify the color assignment of the two varieties (e.g., green to red).

For small and simple experiments, it is possible to identify the optimal design through exhaustive enumeration of all possible designs. However, for more complex experiments, a naive search becomes infeasible, because the number of all possible designs grow exponentially with increasing number of varieties or slides. For example, for 10, 11, and 12 vertices, there are about 11 million, 1 billion, and 150 trillion non-isomorphic connected graphs, respectively. Therefore, the search for near-optimal designs ultimately relies on either following some general guidelines for constructing such designs, or heuristically sampling the search space of all graphs.

In this study, we have developed an effective hill-climbing strategy to search for the near-optimal designs, and harvest the results of the search efforts into a database of near-optimal designs. We have also developed an improved construction algorithm for the interwoven loop designs, which were previously found to be near-optimal [4], but for which no efficient method was present.

## Construction and content

A design matrix corresponding to a microarray design graph *G *is an *n*-by-*m *matrix *X*, where *n *is the number of microarray slides and *m *is the number of varieties. Each row of the design matrix specifies the hybridization used for the corresponding slide, such that the variety labeled with Cy3 is denoted with a 1 and the variety labeled with Cy5 is denoted with a -1. The design matrix for the experiment in Figure 1 is as follows:



Given a design matrix X, an optimality criterion tries to summarize the precision of the parameter estimates in a single score. Differences in defining this precision have given rise to multiple forms of optimality criteria, with A, L, and D optimality being the most common. In MicroarrayDesigner, we define these optimality criteria such that an optimal design would be one that minimizes the corresponding criterion:

• A-optimality is defined as the average variance of the parameter estimates:



• L-optimality is defined as the variance of the parameter estimates with respect to all parameter contrasts *C*:



• D-optimality is defined in terms of the determinant of the design matrix.



Note that the definitions above are only trivially different from their conventional definitions in the literature [4,5]. Particularly, we have scaled the original definition of D-optimality using its logarithm, for numerical convenience. This modification preserves the ordering of optimality scores of different designs.

### Hill Climbing optimization

For a given set of experimental constraints, we would like to find the experimental design that is optimal, i.e., that minimizes the given optimality criteria. The experimental constraints are the number of varieties being analyzed (*n*) and the number of slides (*m*) available for the experiment. Our heuristic search method is based on a hill-climbing approach that seeks to improve a given initial experimental design at each step. This is achieved by repeatedly adding and removing edges (slides) until no further improvement in the optimality criteria can be achieved. The basic algorithm is outlined in Appendix 1.

The algorithm takes an initial design graph *G *= ⟨*V, E*⟩, where *V *and *E *are the list of vertices and edges, respectively. At each iteration, a random number *r *of edges are added one by one. The AddBestEdge function tests all edges (for large graphs, random sampling of edges is performed) and identifies the candidates that improve the optimality criteria of the design the most. These candidates are further filtered with the objective of minimizing the variance in the degree of the vertices, and the distances between pairs of vertices. An edge is randomly selected from the final set of candidates and added to the graph. Similarly, the RemoveWorstEdge function first identifies candidate edges that can be removed with the least degradation to optimality, and a randomly selected candidate edge is removed from the graph. The search algorithm stops when a predefined *maxiter *number of iterations is reached, or when no improvement is obtained for *maxidle *iterations.

### Interwoven Loop designs

Because of the difficulty of analytically or numerically finding the optimal designs, there have been efforts to identify certain recipes for construction of experimental designs. The "reference" and "loop" designs are two such basic types of designs and are the most widely used experimental layouts. In the reference design, each variety is compared to a common reference variety [6]; whereas in the loop design, the varieties are compared to one another in a circular or multiple-pairwise fashion [3]. The loop design is shown to be generally more efficient than the reference design [7,8].

Wit et.al. [4] introduced Interwoven Loop design layouts as ordinary loop designs where each variety was also compared to the varieties that are *j*_2_, *j*3,...,*j*_*n*-1 _'jumps' further along the circle. Interwoven Loop designs were not only shown to be more optimal than the alternative reference and loop designs, but they were also shown to be near-optimal; i.e., achieving an optimality that is close to the theoretically best possible design. However, to the best of our knowledge, no efficient algorithm was hitherto present for the construction of such designs. The size of the class of such designs explored by smida software package [4,9] is exponential in the number of slides and becomes infeasible to compute for large experiments.

As part of the MicroarrayDesigner, we have developed an efficient construction algorithm based on the observation that the jumps in the optimal interwoven loop designs are organized in such a way that the pairwise distances between the nodes are minimized. This heuristic construction (Heuristic Loop) has made it feasible to generate interwoven loop designs for very large experiments. For example, for 10 varieties and 100 slides, it takes the smida program over 10 minutes to find the optimal interwoven loop design, whereas it takes Heuristic Loop under 0.01 seconds to find the same design. For 10 varieties and 200 slides, smida is unable to execute due to excessive memory allocation (with estimated runtime of more than 110 years), whereas Heuristic Loop executes only 0.12 seconds.

### The database and web interface

The nondeterministic search methods generate different results each time they are executed, and it would be a waste of computational time and resources if one did not store the best designs found. The efficient and scalable methods developed in this study have allowed us to compile a database of near-optimal designs for variety and slide numbers of up to 100, which we believe to be a good limit for practical experiments. In order to continually improve the database, a background process keeps searching for better designs, and updates the database designs accordingly. Daily snapshots of the database are made available through the web interface. This database can serve as a practical reference for the biologists, and as a benchmark dataset for research in microarray design.

**Figure 2:**
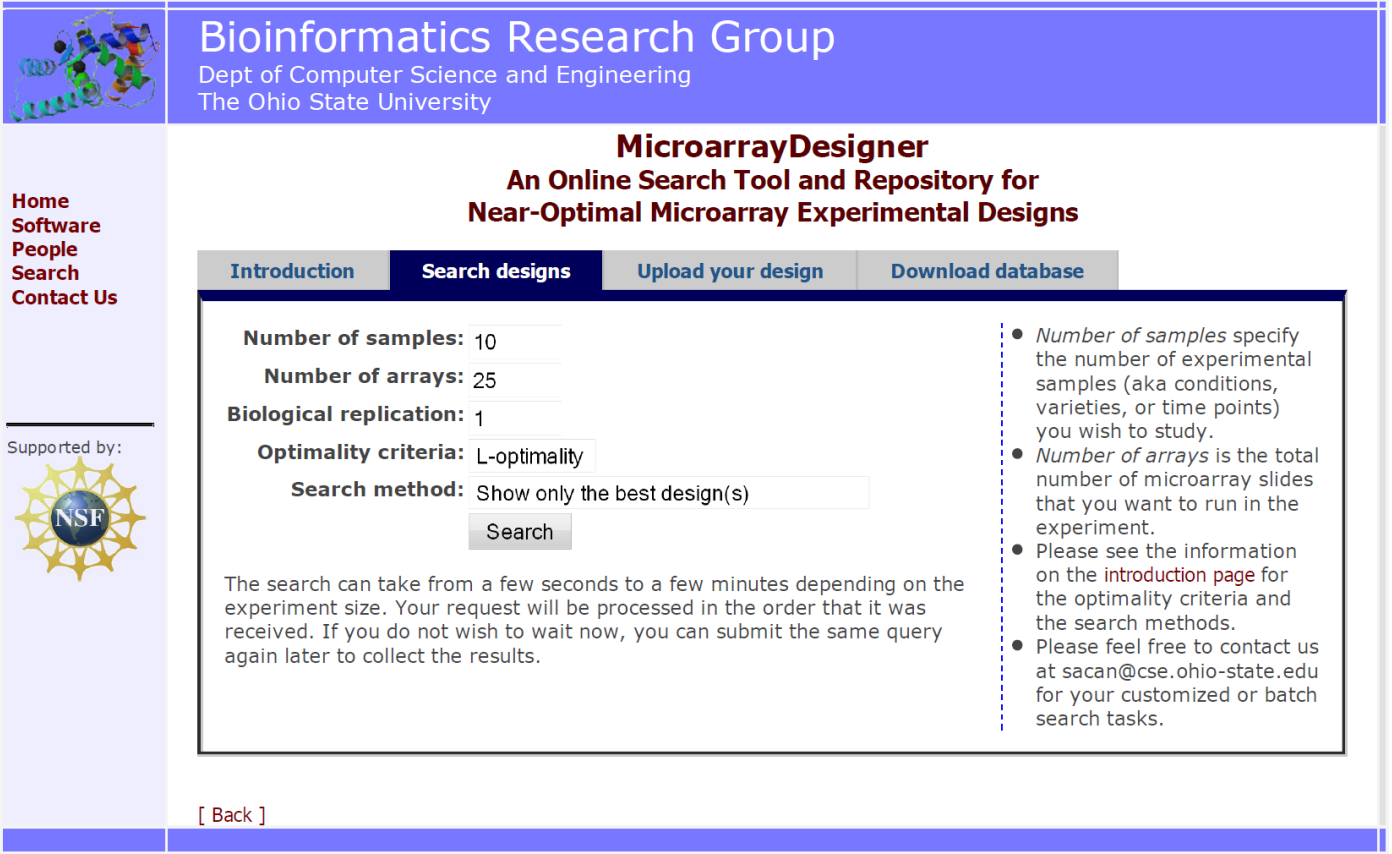
**Screenshot of the MicroarrayDesigner search interface**. The web interface provides a simple search form that allows the user to specify number of varieties, arrays, and biological replication and to select optimality criteria and search method. The user can also upload microarray designs or download a snapshot of the database of designs.

A search interface is provided to allow the users browse available designs, or trigger new searches for experiments that are not already covered by the database (see Figure 2 for a screenshot). The user can select from available methods, and optimality criteria of interest. For small experiments (number of varieties less than 30), the search results are drawn as graphs whose vertices are located around a unit circle. For larger experiments, the graph layout is determined using topological sorting based on the degrees of the vertices. The individual experiment designs can be downloaded as plain-text files. The users are also encouraged to upload their own designs either for comparison with the database designs, or to contribute into improvement of the database.

## Utility

We have tested the methods described above on a large number of test cases, with varying experiment sizes and optimality criteria. Table 1 shows the L-optimality values of the designs found by different methods for a representative sample of these test cases. The results were compared with the Simulated Annealing (smidaSA) method developed by [4]. For smidaSA and Hill Climbing search methods, the tabulated results are the averages of 100 runs. Note that the interwoven loop design is available only when number of slides is an exact multiple of number of varieties.

**Table 1:** L-optimality of designs found by different methods.

**Varieties**	**Slides**	**smidaSA**	**Heuristic Loop**	**Hill Climbing**
5	10	4.22	**4.10**	**4.10**
10	30	14.39	N/A	**14.22**
20	50	86.27	N/A	**83.53**
20	80	49.47	**47.51**	47.75
30	100	146.54	N/A	**141.36**
50	100	861.42	893.63	**825.81**

Each row shows the L-optimality values of the microarray designs found by different methods for selected numbers of varieties and slides. Best results for each row are shown in bold.

For each test case, the Hill Climbing search method found either the best or close to the best design. We attribute the performance of the Hill Climbing to the fact that unlike the random changes employed in the Simulated Annealing method, the changes at each iteration of our algorithm guide the search to a more optimal design. Notably, a re-implementation of the smidaSA with the Hill Climbing search method incorporated as one of the possible steps gave slightly better results than the original Simulated Annealing method. The results of the Hill Climbing-enhanced Simulated Annealing, and of the other test cases are available on the website as part of the database.

## Discussion and conclusion

We have developed an efficient heuristic method for finding near-optimal microarray experimental designs. The proposed method employs a directed hill-climbing algorithm that guides the search toward optimal designs. We have also developed a constructive algorithm for the class of interwoven-loop designs, making construction of these designs feasible for large experiments.

The improved search algorithms have allowed us to generate and maintain continually evolving database of near-optimal microarray experimental designs. This compilation can serve as a reference and benchmark for experiment designers and design optimality researchers. An interactive web interface is provided to query the set of designs for various optimality measures or to upload user-contributed designs. Daily snapshots of the database are also provided for download.

While the early microarray design studies focused on fixed effects models, there have been recent efforts to address the hierarchical or factorial nature of the experimental designs using mixed effects models [2,10]. We remark that the design optimization procedure introduced in this study can not directly be applied to general factorial designs. Nevertheless, in the current version of MicroarrayDesigner, we have implemented a limited support for hierarchical designs with only two levels of factors. Following Ankenman et.al. [11], we have modeled biological replication using nested random factors. Support for a more comprehensive mixed effects model and analysis of the data generated from various experimental designs are out of scope of the current study and are left for future work.

## Availability and requirements

The search tool and the database are accessible via the World Wide Web at . The source code and binary distributions for the search algorithm and the web service are available from the authors for academic use. Computation of the optimality criteria and the search algorithms are implemented in MATLAB. The database of experimental designs is stored as plain text files to simplify distributed processing and to allow direct packaging of the database for download.

## Authors' contributions

NF and HF conceived of the study and participated in the coordination of the project. All authors participated in the design of the study and development of the algorithms. AS and NF implemented the search algorithms and performed the computational experiments. All authors participated in the analysis of the results. AS developed the database and the web interface. AS and NF contributed to the writing the manuscript. All authors read and approved of the final draft.

## Appendix 1 - The Hill Climbing optimization algorithm

The algorithm optimizes an input design graph *G *by repeatedly adding and removing a random number of edges each of which improve the optimality criteria. *AddBestEdge *iterates over each pair of nodes in the graph and adds the edge that results in the highest increase in optimality. Likewise, *RemoveW orstEdge *iterates over each of the existing edges and removes the one that results in the highest increase in optimality. The procedure is repeated a specified *maxiter *iterations or until no improvement is achieved over *maxidle *iterations.

   **Input**: initial design graph *G *= ⟨*V*, *E*⟩

   **Output**: optimized design *G*

   **for ***i *← 1 **to ***maxiter ***do**

      *G*' ← *G*;

      *r *← rand * |*V*|;

      **for ***j *← 1 **to ***r ***do**

         AddBestEdge(*G'*);

      **for ***j *← 1 **to ***r ***do**

         RemoveWorstEdge(*G'*);

      **if ***optimality did not improve past maxidle iterations ***then**

         break;

      *G *← *G*';
